# Recombinant Endostatin as a Potential Radiosensitizer in the Treatment of Non-Small Cell Lung Cancer

**DOI:** 10.3390/ph16020219

**Published:** 2023-01-31

**Authors:** Charnay Cunningham, Julie Bolcaen, Alessandra Bisio, Amanda Genis, Hans Strijdom, Charlot Vandevoorde

**Affiliations:** 1 Centre for Cardio-Metabolic Research in Africa (CARMA), Division of Medical Physiology, Stellenbosch University, Cape Town 7602, South Africa; 2 Radiation Biophysics Division, SSC Laboratory, NRF Ithemba LABS, Cape Town 7131, South Africa; 3 Department of Cellular, Computational and Integrative Biology—CIBIO, University of Trento, 38123 Trento, Italy; 4 Biophysics Department, GSI Helmholtzzentrum für Schwerionenforschung, Planckstr. 1, 64291 Darmstadt, Germany

**Keywords:** endostatin, endostar, radiation therapy, proton therapy, anti-angiogenic therapy, vascular endothelial growth factor (VEGF), VEGF receptor inhibitor, tumour Angiogenesis

## Abstract

Non-small cell lung cancer (NSCLC) is the most prevalent type of lung cancer, which is the leading cause of cancer-related deaths worldwide. Over the past decades, tumour angiogenesis has been intensely studied in the treatment of NSCLC due to its fundamental role in cancer progression. Several anti-angiogenic drugs, such as recombinant endostatin (RE), have been evaluated in several preclinical and clinical trials, with mixed and often disappointing results. However, there is currently an emerging interest in RE due to its ability to create a vascular normalization window, which could further improve treatment efficacy of the standard NSCLC treatment. This review provides an overview of preclinical and clinical studies that combined RE and radiotherapy for NSCLC treatment. Furthermore, it highlights the ongoing challenges that have to be overcome in order to maximize the benefit; as well as the potential advantage of combinations with particle therapy and immunotherapy, which are rapidly gaining momentum in the treatment landscape of NSCLC. Different angiogenic and immunosuppressive effects are observed between particle therapy and conventional X-ray radiotherapy. The combination of RE, particle therapy and immunotherapy presents a promising future therapeutic triad for NSCLC.

## 1. Introduction

### 1.1. Treatment Landscape of Non-Small Cell Lung Cancer

Globally, lung cancer is the second most commonly diagnosed cancer and leading cause of cancer related deaths [1]. Non-small cell lung cancer (NSCLC) is the most prevalent type of lung cancer making up about 85% of all lung cancer cases [2]. Based on histological features, NSCLC can be further subdivided into squamous cell carcinoma, large cell carcinoma, and lung adenocarcinomas [3]. The five year survival rate for patients with localized NSCLC is 64%, however, most patients already present advanced disease with local progression and metastasis at first diagnosis, leading to an overall five year survival rate of approximately 15% for all NSCLC stages [4,5,6]. Depending on the stage, histology, genetic alterations, and the general condition of the patient, the standard treatment of NSCLC usually includes surgery, chemotherapy, radiotherapy (RT), targeted therapy or immunotherapy, either alone or in a combined treatment regimen [7]. The latest advances in systemic treatment have been driven primarily by the development of molecularly targeted therapeutics, immune checkpoint inhibitors, and anti-angiogenic agents. Tyrosine kinase inhibitors are now approved to treat several subtypes of NSCLC [7]. Chemotherapy for NSCLC consists of a combination of platinum-based drugs and cytotoxic drugs like paclitaxel [7]. A well-known example of an anti-angiogenic drug that reached the clinic for NSCLC patients is an antibody against the vascular endothelial growth factor (VEGF)-A, bevacizumab. It has been approved as a first-line treatment for advanced-stage patients in combination with platinum-based chemotherapy [8,9]. Until today, extensive research has been performed on anti-angiogenic drug candidates for the treatment of NSCLC patients. This review will focus specifically on Endostatin, a promising drug for NSCLC in combination strategies. However, this narrative review will also clarify that more work is needed to identify predictive biomarkers and main angiogenic role players in order to optimise angiogenic therapy, particularly in the context of combination treatments for NSCLC. 

### 1.2. The Role of Tumour Angiogenesis in NSCLC

During the early stages or dormant phases of cancer development, tumours can exist without blood supply for extended periods of time (months to years) [10]. However, the absence of tumour vasculature becomes a critical determinant during tumour progression. Adequate delivery of nutrients and oxygen are a significant requirement to meet the metabolic demands of a growing tumour [11]. In addition, as the neoplasm enlarges, apoptotic and necrotic zones are created. This leads to the presence of hypoxia in the growing tumour mass, which is the vital initial stimulus for tumour vascularisation and the initiation of angiogenesis [12]. While angiogenesis is a tightly regulated process under normal physiologic conditions, the vascular network created in tumour angiogenesis is markedly disordered and dysfunctional, resulting in increased vascular permeability [13]. 

The tumour angiogenesis process requires an imbalance between the different major pro- and anti-angiogenic factors, which have been extensively studied and defined over the past decades [14]. This imbalance is also coined as the “angiogenic switch” in tumours and is characterized by the promotion of a pro-angiogenic milieu [15,16,17]. The key signalling process in the development of the tumour vasculature is the hypoxia-induced stimulation of Hypoxia Inducible Factor 1 alpha (HIF 1-α), resulting in the suppression of anti-angiogenic factors, such as trombospondin-1 and angiostatin, whilst simultaneously promoting the production of Vascular Endothelial Growth Factors (VEGF) and basic fibroblast growth factor (bFGF) [18]. The latter are known as positive regulators of angiogenesis, promoting vascular permeability and endothelial cell proliferation, respectively [19]. The VEGF family comprises 5 VEGF glycoproteins, namely VEGF-A to D, and placental growth factor 1 and 2 (PIGF-1 and -2) [20]. They exert their biological action by activating tyrosine kinase receptors and VEGF receptors (VEGFR) 1 and 2 present on vascular endothelial cells [15]. Together with VEGFs, angiopoietins co-regulate angiogenesis. In particular, Ang-1 and Ang-2 bind to Tie-2 receptors to control blood vessel stabilization signals [21]. Alternatively, in the event of their blockade, the roles of VEGFs and angiopoietins can be assumed by bFGF. The FGF family interacts with four main FGF receptors (FGFRs), and the FGF ligands are involved in promoting proliferation, survival, migration, proteinase production, and the expression of specific integrins in vascular endothelial cells [22]. It is important to note that FGFs have been reported to promote angiogenesis independently from VEGF and potent angiogenic activity has been identified for FGF-2 [19,22]. Therefore, the signalling pathways of FGF/FGFR as well as the platelet-derived growth factor (PDGF) and PDGF receptor (PDGFR), could provide potential escape mechanisms from anti-VEGF/VEGFR therapy that could facilitate resumption of tumour growth [23]. Undeniably, complex and overlapping signalling cascades govern tumour angiogenesis and cause resistance to anti-angiogenic therapy [24,25,26,27,28,29]. In addition, once the tumour angiogenesis process has been activated, tumour metastasis can also occur via these newly formed vessels [4]. 

In NSCLC specifically, HIF-1α and HIF-2α are commonly overexpressed, correlating with poorer survival and increased microvascular density (MVD), respectively [30,31]. Work by Lin and colleagues provided evidence that high HIF-1α expression is an independent prognostic factor of small cell lung cancer (SCLC) with a 39.2 fold risk of mortality [32]. Tumour angiogenesis has also been identified as a crucial prognostic factor in advanced NSCLC. For example, the level of angiogenesis (high MVD) was associated with shorter survival of patients with stage IIIA NSCLC [32]. The use of MVD as a prognostic factor remains a controversial subject in NSCLC, mainly due to the large variation in different histological studies regarding tumour growth patterns and different definitions of angiogenic profiles and prognosis [33]. Although the traditional angiogenic processes govern tumorigenesis in NSCLC, it also exhibits unique features, such as vessel co-option (VCO) [34]. In this instance, the tumour growth also relies on its invasion of the host tissue. In fact, it was possible to classify NSCLC according to its morphological features, based on the biological characteristics of the tumour-lung interface [35]. This classification of Sardari et al. distinguishes three distinct NSCLC growth patterns: (a) a destructive growth pattern, which entails a traditional angiogenic growth pattern; (b) the papillary growth pattern that involves the preservation of the alveolar structure of the lung parenchyma at the interface with co-option of alveolar blood vessels with stromal stalk formation and subsequent angiogenesis. Finally, there is (c) the alveolar growth pattern; wherein the alveolar structures of the lung parenchyma are preserved, and co-option of septal blood vessels occurs without evidence of new stroma formation at the interface [35]. Recently, Cuypers and co-authors described VCO in great detail and identified it as a new placeholder in the assessment of tumour vasculature [36]. However, compared to traditional tumour angiogenesis, the molecular events governing VCO remain largely understudied. Consequently, no treatment strategies exist to inhibit VCO. New role players in the angiogenic progression of NSCLC have been described, such as sex dependency and specific non-coding RNAs [32,37]. It is clear that the angiogenic landscape in NSCLC is multifaceted, and various efforts in mechanistic investigation and treatment strategy remain necessary.

### 1.3. The Potential of Anti-Angiogenic Drugs in Combination Treatments for NSCLC

Since tumour angiogenesis was first proposed in the pioneering work of Folkman in 1971 by the discovery of the first angiogenic factors, anti-angiogenic drugs have been earmarked as a promising cancer strategy [38]. With the more precise classification of angiogenic processes and signalling pathways, targeted therapies evolved with the aim to block tumour angiogenesis [39]. They would inhibit tumour blood vessel production, leaving cancer cells in starvation by blocking the supply of oxygen and nutrients, which could subsequently lead to the creation of hypoxic areas in the tumour [40,41]. The presence of hypoxia is particularly important in the context of RT, where it can reduce the radiosensitivity of the tumour cells. Therefore, it sounds counterintuitive to inhibit tumour angiogenesis to improve the efficacy of RT to obtain tumour control [42]. However, the rationale lies in the use of anti-angiogenic therapies to ‘normalize’ the tumour vasculature by pruning the immature and inefficient blood vessels [13]. This could eventually lead to a normalized vasculature, which is more conducive to the delivery of targeted drugs as well as a net increase in tumour oxygen concentration, which can increase the effectiveness of RT. The latter will be discussed in Section 3 of this review. Besides, the concept of vascular normalization is also receiving growing attention in combination with immunotherapy, with or without the addition of RT [43,44,45,46,47]. 

This is particularly important for NSCLC, known as a highly vascularized tumour, where histological evidence of enhanced angiogenesis has been associated with poor prognosis [48,49]. As a result, anti-angiogenic therapy for NSCLC was already tested at the beginning of this century [8]. Unfortunately, only marginal benefit was observed in early clinical trials with vessel-inhibiting therapies for advanced NSCLC, where the focus was mainly on the repression of vessel sprouting by inhibition of VEGF signalling [50]. Multiple trials also explored the combination of bevacizumab with tyrosine kinase inhibitors (TKIs), but the optimal sequence for administration of these drugs remains to be determined [51]. Other anti-angiogenic agents, such as sunitinib, sorafenib, and vandetanib, have proven to be unsuccessful in clinical trials, while two new anti-angiogenic agents (ramucirumab and nintedanib) produced a significant survival benefit in a second-line setting [9]. The limited clinical efficacy is most likely attributable to alternative processes in the tumour microenvironment (TME) which are resistant to traditional angiogenesis inhibitors. Specific sub-groups of NSCLC with non-angiogenic patterns have been described, where tumours seem to co-opt the existing blood and lymphatic vessels via a process called VCO (as described in Section 1.2), rather than inducing angiogenesis [34,52]. Another non-angiogenic mechanism is termed vasculogenic mimicry and is based on the self-organizing ability of cancer cells into vascular-like structures, allowing them to obtain nutrients and oxygen autonomously [53]. These alternative non-angiogenic processes in NSCLC progression lead to resistance to VEGF-inhibitors and contribute to therapy failure [54]. Furthermore, it is unlikely that the inhibition and control of the tumour vasculature as a stand-alone therapy will cure a cancer, but it has the potential to limit its growth and spread. More importantly, it can also potentiate the effect of direct anti-tumour therapies, such as standard chemotherapy and RT [14]. 

In this review, we will focus on a drug that was inspired by one of the first anti-angiogenic factors to be discovered, namely endostatin, produced by murine haemangioendothelioma cells in Folkman’s laboratory [38]. It is one of the most effective endogenous inhibitors of angiogenesis, proven to be a promising tool to inhibit at least 65 different tumour types [55,56,57]. As soon as recombinant endostatin (Endostar®, further referred to as RE) was generated in a stable and soluble form that was cost-effective to produce, the drug was subsequently tested for the treatment of many cancer types [58]. RE was found to be more stable with a longer half-life than bevacizumab and inhibited tumour vascular growth through multiple targets [59]. Extensive research has been performed on the chemo- and radiosensitising effects of endostatin [58,60,61,62,63,64,65]. This led to its approval by the State Food and Drug Administration (FDA) of China in 2005 for the treatment of NSCLC [58]. More recently, it has also been approved by the USA FDA for the first or second line treatment of NSCLC [66]. This review will focus on the current status of endostatin/RE for the treatment of NSCLC, with a focus on the combined use with RT to overcome treatment resistance.

## 2. Endostatin and Its Mechanism of Action

**Figure 1:**
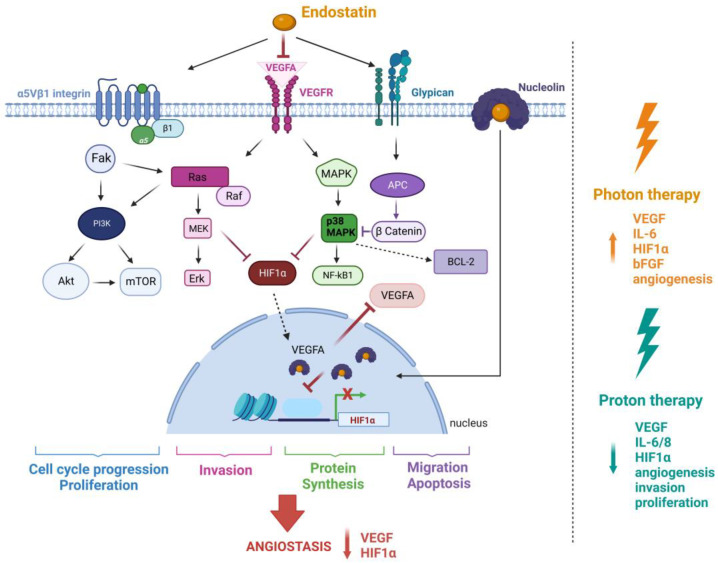
The The mechanism of action of RE and the effect of photon-based radiotherapy and proton therapy (PT) on tumour angiogenesis. RE inhibits VEGF-A binding to VEGFR-1/2 resulting in the inhibited activation of kinase/c-Raf/MEK1/2/p38/ERK1 MAPK pathway. It exerts dual suppression on the PI3K-AKTpathway by binding to the receptor α5β1 integrin. RE’s low affinity glypican binding leads to Wnt pathway signalling disruption and β-catenin degradation. The association of RE with nucleolin and subsequent nuclear translocation inhibits the transcription of HIF-1α resulting in decreased VEGF-A production. Endostatin also increases apoptosis in endothelial cells by the downregulation of the anti-apoptotic protein Bcl-2. Different cellular mechanisms corresponding to various signalling pathways are consequently inhibited. Synergy with radiotherapy is dependent on the radiation quality. Photons are known to promote angiogenesis and cause an increase in the expression of pro-angiogenic factors, such as vascular endothelial growth factor (VEGF), Interleukin 6 (IL-6), Hypoxia inducible factor 1 alpha (HIF-1α), and basic fibroblast growth factor (bFGF). In contrast, proton irradiation significantly downregulates some of the same and other pro-angiogenic factors, resulting in the inhibition of tumour angiogenesis. Figure was created with BioRender.

Endostatin is a C-terminal fragment cleaved from the NC1 domain of Collagen XVIII via the proteolytic activity of proteinases like elastase, procathepsin L, and matrix metalloproteinases (MMPs) [67]. Several animal studies demonstrated that endostatin had the ability to suppress neovascularization, resulting in growth inhibition in several murine and human tumours [68,69]. Endostatin and RE are known as broad-spectrum angiogenesis inhibitors, which interfere mainly with the pro-angiogenic function of VEGFs and FGFs. Nucleolin, a cell surface phosphoprotein constituent, also acts as a receptor for endostatin. Upon binding to RE and its heparin-binding site, nucleolin is internalized and translocated to the cell nucleus, shuttling RE along with it (Figure 1) [70]. The abundance of nucleolin receptors on endothelial cell surfaces gives RE the ability to stall the migratory action of endothelial cells [71]. In addition to the inhibition of endothelial cell migration, RE also inhibits proliferation, induces cell cycle arrest, and stimulates endothelial cell death by apoptosis [72,73]. Furthermore, the interaction of RE with endothelial cells results in the activation of a variety of downstream effects, such as the inhibition of the Wnt/β-catenin pathway and an actin reorganization in endothelial cells [74]. However, despite the growing number of clinical trials, the mechanism of action of RE has proven to be more complex than initially surmised. In addition to its binding efficiency with nucleolin, endostatin interferes with several processes and is also known to interact with various other receptors, such as VEGFR-2 and -3, glypican 1 and 4 and integrin v5 and α5 receptors. The interaction between RE and integrins is also related to the disruption of cell migration by outcompeting the binding of the pro-angiogenic ligand fibronectin to α5β1 integrin, which would promote cell migration [75]. RE suppresses the VEGF-induced tyrosine phosphorylation of kinase insert domain containing receptor/fetal liver kinase 1 (KDR/Flk-1/VEGFR-2) as well as the overall VEGFR-2 expression and the activation of extracellular signal related kinase (ERK), p38 mitogen-activated protein kinase (p38 MAPK), and Protein kinase B (AKT) in human umbilical vein endothelial cells (HUVECs) (Figure 1) [76]. It is also suggested that both heparan sulphate (HS) and α5β1 integrin need to be present for the localization of endostatin in endothelial cell lipid rafts [77]. The association between HS and integrin was proven to lead to the inhibition of focal adhesion kinase c-Raf-MAPK pathway, showing similar downstream suppression effects to VEGF-A binding to endostatin, ultimately leading to the inhibition of endothelial migration [75].

Molecular studies have reported that RE can induce the attenuation of focal adhesions, a functional protein complex that links the actin cytoskeleton of the endothelial cells to the underlying basal membrane in human dermal microvascular endothelial cells [78]. In contrast, RE increased the number of focal adhesions in bovine capillary endothelial cells. These increases in focal adhesions were sustained with the administration of FGF-2. In contrast, RE has also been proven to disrupt cytoskeletal arrangement in addition to cell-cell matrix interactions [79].

RE also possesses ATPase activity, which led to the development of an engineered form of endostatin, which exhibits much higher ATPase activity than the wildtype one [75]. It was shown that the ATPase activity of RE is required to inhibit the action of tumour-associated macrophages (TAM) [80]. This is an intriguing finding since TAMs can enhance tumour angiogenesis, immunosuppression, tumour cell invasion and metastasis. Furthermore, their association with angiogenesis and lymphangiogenesis, contributes to the progression of NSCLC [16,81]. 

Collectively, these studies fall under a large blanket of experimental findings that show RE’s effect on the vascular endothelium and successfully served as a validation of the original work by O’Reilly and colleagues [38]. Importantly, anti-angiogenic drugs like RE target rapidly proliferating tumour-associated endothelial cells rather than relatively dormant endothelial cells in healthy tissue, making them less toxic than chemotherapeutic agents [82].

### RE Re-Imagined

The formulation of RE, a protein drug, is coupled with limitations such as poor bioavailability, insoluble and unstable nature, and high production cost. Therefore, several attempts were made to improve RE structurally, that include PEGylation of its N-terminus, the addition of an RGD (Arg-Gly-Asp) sequence that is present in integrin ligands, fusing endostatin to the Fc region of IgG, or the addition of Zinc [83,84,85,86]. An alternative to RE is the introduction of human endostatin cDNA via viral and non-viral vectors [87,88]. In a phase I dose-escalation clinical trial for multiple cancer types, the intra-tumoural injections of an adenovirus containing the human endostatin gene resulted in a decrease in bFGF expression levels and angiogenic serum markers [87].

RE-loaded nanoparticles have also shown anti-angiogenic effects in vivo; for example, a folic acid-decorated chitosan nanoparticle successfully targeting squamous cell carcinoma (SCC) [89,90,91]. VEGFR-2 was successfully targeted in the blood brain barrier by Lu et al., using a dual receptor peptide functionalized polyethyleneimine nanocomplex for secretory RE delivery to malignant glioma [92]. Gold nanoparticles (rHES-AuNPs) were utilized by Li and colleagues to normalize vasculature in NSCLC and improve chemotherapy in mice bearing H22 tumours [93]. Interesting work has also been performed with RE liposomal formulations. Liposomal encapsulation of RE resulted in increased stability and half-life of the peptide as well as gradual release of the peptide. [94]. RE loading into liposomes has also been used in a nanoformulation that was used as a mode of gene therapy with strong anti-tumour effects in vivo [95]. Nanoformulations of RE have also been studied in combination with RT [96]. Using hyaluronic acid-tyramine as a carrier, an ES-loaded hydrogel drug (ES/HA-Tyr) was synthesized for local injection [96]. The ES/HA-Tyr formulation increased local drug concentration, decreased blood drug concentration, and caused less systemic toxicity in an in vivo study design. Additionally, ES/HA-Tyr effectively reduced tumour microvessel density, increased tumour pericyte coverage, decreased tumour hypoxia, and increased RT response [96]. Although the pre-clinical work in nanomedicine was promising, the clinical translation of nano-incorporated drug formulations has been hindered by the lack of patient overall survival improvement, suboptimal nanoparticle biodistribution, and safety concerns [97]. 

## 3. Radiotherapy and Anti-Angiogenic Therapy: A Dilemma

**Figure 2:**
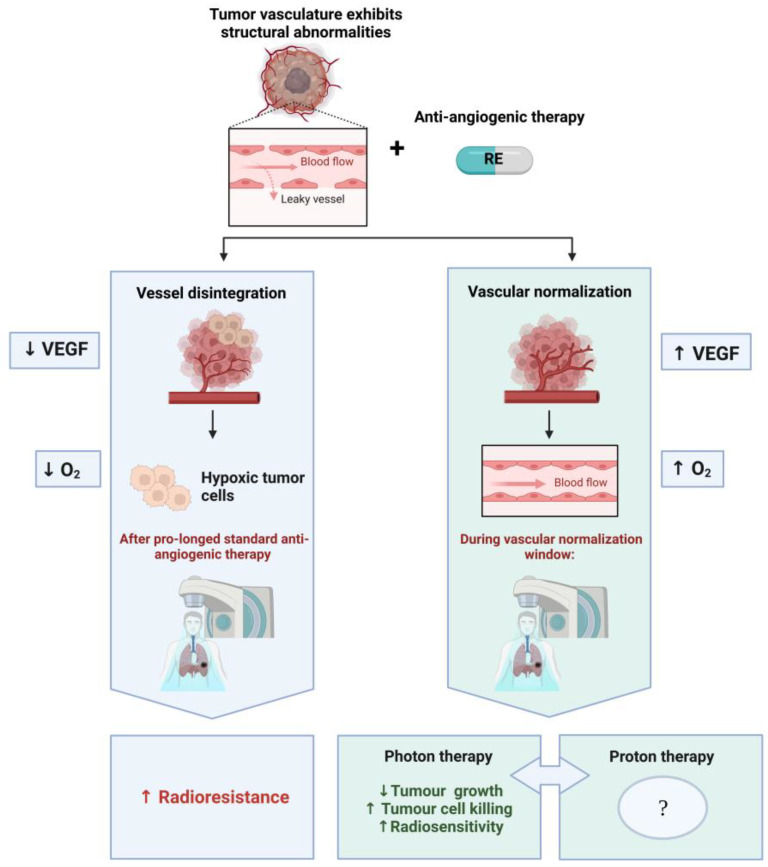
The paradoxal combination of RE and RT. The left-hand side (blue) illustrates the initial approach, where anti-angiogenic therapy blocks the formation of blood vessels and enhances hypoxia within the tumour, due to the reduction of blood vessels, resulting in increased radioresistance during RT. If one administers RE at a well-defined dose, a normalization window will appear at a given time (variable timing depending on tumour type, administration route, and dose). In this case, the RT can benefit from vascular normalization (right column—green), which induces the conversion of a chaotic to a more normal vascular network in the tumour and results in an enhancement of tumour oxygenation and radiosensitivity. This contributes to increased tumour control and tumour cell killing in experimental studies with X-ray (photon)-based RT. No preclinical or clinical studies are currently available on the combination of RE with proton therapy, which calls for further investigation to decipher the combined effects. Figure was created with BioRender.

As previously mentioned, the administration of anti-angiogenic therapy as a radiosensitizer seems paradoxical. Blocking the formation of blood vessels could enhance hypoxia within the tumour and contribute to increased radioresistance (Figure 2) [98,99]. This principle is based on the oxygen effect, one of the pillars of the “five Rs” of radiation biology, which form the basis of RT [100]. When low linear energy transfer (LET) radiation is used, such as high-energy (MV) X-rays, the presence of oxygen will “fixate” the DNA radicals, which are a result of the reaction between the DNA and the hydroxyl radicals produced in the surrounding water [101]. The excessive reduction of vessels by anti-angiogenesis therapy has been shown to cause additional intra-tumoural hypoxia resulting in pathological angiogenesis, inflammation, increased migration, and additional sequelae [102]. However, the theory of vascular normalization, initially proposed by Jain et al. [103], is based on the hypothesis that anti-angiogenic treatment can revert the structurally and functionally abnormal tumour vasculature toward its normal state (Figure 2) [104]. If treated with the appropriate treatment dose, this results in the conversion from a chaotic to a more ‘normal’ vascular network in the tumour. In short, this leads to reduced vascular permeability and interstitial fluid pressure, improved blood flow and increased tumour perfusion, and consequently, a reduction of tumour hypoxia. This effect can enhance the systemic delivery of cytotoxic drugs, immunotherapy, and improve tumour radiosensitivity [102,105] The concept was elegantly demonstrated by Lee and colleagues for RT, where an enhanced tumour response was observed by combining radiation with an anti-VEGF monoclonal antibody [106]. Tumour growth inhibition was accompanied with a significant reduction in tumour vasculature density, a decrease in interstitial fluid pressure, and an increase in partial oxygen tension. The most complex part is defining the “normalization window” since vessel normalization is transient and hard to capture, despite its recognisable pattern (e.g., vascular structure plasticity and changes to the tumour microenvironment) [13,107,108]. Vascular normalization occurs very quickly in both human and murine models, often within a day and it lasts approximately 1 week to a couple of months in humans [109,110]. These effects are also limited spatially and temporally and differ for diverse types of cancers. Predictive detection of microvessel architectural parameters could be based on Magnetic Resonance Imaging (MRI), Vessel Architectural Imaging (VAI), Microvascular Density (MVD), or Positron Emission Tomography (PET) [108,110]. 

In addition to the dilemma created by the normalization window and the optimal timing between drug administration and fractionated RT, anti-angiogenics have not been investigated in combination with particle therapy. This is a rapidly growing field within RT, where particles such as protons and carbon-ions are used which present physical and radiobiological advantages over conventional high-energy X-rays to treat cancer [111,112,113]. Furthermore, contrasting radiobiology reports exist on the tissue-level effects of X-ray based RT versus particle therapy, which could significantly impact disease progression, such as angiogenesis [114,115,116]. A growing number of studies highlights the differences between X-rays and particle beams on pro- and anti-angiogenic effects (Figure 1). Kamlah and collaborators explored the angiogenic effects of carbon ions and X-rays in A459 tumour bearing BALB/c *nu/nu* mice [117]. The A549 cells were irradiated with both radiation types and injected in the mice to generate a plug, allowing the quantification of blood vessel formation. A significant increase in blood vessel density was observed after X-ray irradiation (6 Gy), but not with carbon ions (biological equivalent dose, 2 Gy) [117]. Takahashi and colleagues reported the inhibition of endothelial cell migration and invasion at sublethal doses of carbon ions, while sublethal X-rays promoted endothelial cell migration and progression of capillary-like tube structures, even after doses as high as 16 Gy [118]. High-energy proton irradiation inhibits multiple angiogenesis-associated processes, including invasion and endothelial cell proliferation [116]. Dose-dependent suppression of angiogenic signalling was demonstrated in both cancerous and non-transformed cells. Additionally, the downregulation of VEGF-A, interleukin 6 and 8 (IL-6, IL-8), and HIF-1α was reported [116]. In contrast, Girdhani reported the upregulation of anti-angiogenic genes like VEGF-A, IL-6, and HIF1α after endothelial cell exposure to high-LET ^56^Fe ion radiation [119]. The radiation quality that is used for therapy will have to be considered in targeting tumour angiogenesis, as well as in the successful incorporation of anti-angiogenics. The lessons learned from X-ray-based RT may not guarantee similar outcomes when incorporated into particle therapy.

### 3.1. RE and Vascular Normalization

The current status and study progress on the normalization window of RE was comprehensively reviewed by He et al. [120]. RE’s effectiveness as an anti-tumour drug has been correlated to its ability to restore vascular normalization and reduce HIF-1α expression and its related signalling pathways [58,76,121]. This observation has led to investigations into RE’s efficacy as a vascular normalizer in various lung cancer models [122,123,124]. Transient vascular normalization occurred in A549 lung adenocarcinoma murine models between 7–10 days after RE administration. Within this same period, an increase in activated circulating endothelial cells, decreases in intra-tumour hypoxia, vessel permeability, and microvascular density were reported [122]. Furthermore, the maximal anti-tumour effects of cisplatin were observed on day 5–9 after the initial administration of RE, improving the synergistic efficacy of RE and cisplatin. In a murine xenograft model of lung cancer, tumour vessels normalized and matured on day 6 of RE-therapy. In addition, the amount of M2-like TAMs in the tumours decreased, whereas the number of M1-like TAMs increased during vascular normalization [123]. An RE-induced decrease in tumour hypoxia after 5 days of RE administration has also been reported in NSCLC patients [124]. Hypoxic alleviation was shown on day 5, both clinically (in patients) and in Lewis lung carcinoma (LLC) models. In mice, the most significant growth delay was observed when RT was given on day 5, which was superior to single therapy with RE, RT or when RT was given 1 day before or after RE [125].

RE’s vascular normalization capability has also been exhibited in other cancer models. In colon and nasopharyngeal carcinoma-bearing mice, RE treatment inhibited vascular endothelial growth and increased pericyte coverage, which led to tumour vascular normalization [126,127]. A normalization window appeared by day 5 to 7, resulting in improved anti-tumour effects of RT [127]. RE also improved the anti-tumour effects of Programmed death ligand 1 (PDL-1) inhibitors in a colon carcinoma model. Furthermore, after 27 days, the effect of RE alone was reported to cause significantly lowered levels of VEGF and transforming growth factor β (TGF-β) [128]. 

### 3.2. Summary of Preclinical Results on RE Combined with RT 

Interestingly, there are less preclinical studies compared to clinical evaluations of native RE in combination with RT. Table 1 and Table 2 summarize multiple studies that investigated the combined effect for different cancer types in vitro (cells) and in vivo (animal models). The most notable in vitro effects caused by RE and RT were cell cycle disruptions [129,130], enhanced cellular radiosensitivity resulting in changes in proliferation, invasion, and migration [130,131,132]. In general, a combination therapy seems to inhibit tumour cell growth and improve the effects of photon-based RT (incl. high-energy X-rays and ^60^Co γ-rays). 

Tumour growth inhibition was often noted in in vivo models (Table 2). Normalized vasculature was observed when RE was combined with photon-based RT, as well as successful tumour regression attributable to improved hypoxic conditions [107,119,126]. Ling and co-workers treated RE gene-transfected lung adenocarcinoma (A549) cells with RT, which synergistically inhibited neovascularization and tumour growth [133]. RE gene-transfected B16 melanoma bearing mice showed marked reductions in intra-tumoural vascularization upon combination with X-rays [134]. At a selected dose of 15 Gy, RE incorporated with ^137^Cs γ-rays showed pronounced tumour growth in mice bearing A431 cell epidermoid tumour xenografts. RE had little to no effect on tumour cell apoptosis over time, while IR alone significantly increased tumour cell apoptosis. RE combined with RT, however, increased tumour cell apoptosis by a factor of two and blocked tumour revascularization [135]. Finally, in both in vitro and in vivo analyses, VEGF/ VEGFR pathway signalling was implicated in the efficacy of the RE and RT combination [132,136]. By examining the VEGFR-2 high-expressing cell line Calu-1 and VEGFR-2 low-expressing cell line A549, Liu et al. showed that RE and RT induced apoptosis and enhanced radiosensitivity in Calu-1 cells, while a limited effect was observed in A549 lung adenocarcinoma cells [132].

As illustrated in Table 1 and Table 2, RE in combination with RT has also be evaluated in preclinical settings for several non-pulmonary cancer types, such as breast, oesophageal, hepatocellular, colorectal and nasopharyngeal carcinoma [127,131,137,138,139,140,141,142,143]. The two in vitro studies on breast cancer cell lines of Aydemir and co-workers confirmed that RE potentiated the anti-tumour effect of RT [138,139]. In their first study, RT alone inhibited the growth of 4T1 (30.81%) and 4THMpc (39.64%) cells, while the addition of RE enhanced the growth inhibition to 83% in 4T1 and 80% in 4THMpc cells [138]. For oesophageal cancer, the in vitro study did not show an enhanced level of apoptosis on the Eca-109 and TE13 cell lines when RE was combined with RT [131]. However, RE combined with radiotherapy significantly inhibited the proliferation, migration, invasion, and vascular mimicry of human oesophageal cancer cells in a dose-dependent manner. The latter showed that the combination of RE with RT has the potential to significantly change the microenvironment of oesophageal carcinoma. In parallel, two in vivo studies illustrated that RE improved the radioresponse in oesophageal xenograft mouse model [139,144]. Both studies showed a reduction in MVD on histological tumour sections and a delay in tumour growth in the treatment groups with RE and RT, compared to RT alone. Both studies did not clearly define the appearance of the vascular normalization window, but Zhu and co-workers reported an improvement of tumour hypoxia 12 days after the start of the RE treatment [141]. In a hepatocellular carcinoma (HCC) bearing mouse model, RT alone increased the expression of VEGF (38.7 ± 5.8), while combination therapy with RE reduced VEGF (15.0 ± 1.8) expression as well as the MVD [142]. The combination of RE and RT showed the highest levels of tumour growth inhibition, while RE alone did not always lead to a higher inhibition effect. The latter study did not specifically determine the normalization window, but administered RE 7 days before the RT, followed by continuous RE treatment after RT to obtain the synergic therapeutic effect. Based on the different preclinical studies, it is clear that more studies are needed to clearly investigate the influence of different RE concentrations and the timing of the vascular normalization window. The administration of RT within this window is critical to obtain a maximal therapeutic effect.

**Table 1:** Summary of in vitro studies on RE combined with radiotherapy.

Cancer Type *Cell Lines*	Endostatin Type *Dose*	Main Result	RT Type *Dose*	Year	Reference
Breast Cancer *4T1 or 4MTMHpc*	RE (murine) *0.5, 1, 2, 4 and 8 µg/mL*	Inhibits the in vitro growth and potentiates the anti-tumour effects of RT via alteration of the amount of substance P	^60^Co γ-rays *45 Gy*	2011	[138]
Human Pulmonary Adenocarcinoma *A549*	RE *300 mg/L normoxia; 400 mg/L hypoxia*	Radiosensitizing effect under hypoxia, but not under normoxia. RE enhanced radiosensitivity through G2/M arrest	6 MV X-ray *2 Gy*	2012	[129]
Human ESCC *Eca109 and TE3*	RE *25, 50, 100, 200, 400, 600, and 800 µg/mL*	Combined treatment inhibited migration, invasion, and vasculogenic mimicry formation, but did not enhance radiosensitivity	6 MV X-ray *2, 4, 6 or 8 Gy*	2016	[131]
NSCLC *Calu-1, A549, 95D, NCI-H292, NCI-H1299*	RE *0, 200, 500, 1000, 2000, and 2500 µg/mL.* *IC20 of Calu-1 cells: 296.5 μg/mL*	Induces apoptosis and enhances radiosensitivity of the VEGFR-2 high-expressing cell line Calu-1, but it has a limited effect on the VEGFR-2 low- expressing cell line A549	not stated *2, 4, 6 and 8 Gy*	2016	[132]
Breast Cancer *4T1 or 4MTMHpc*	RE *0.5, 1, 2, 4 and 8 µg/mL: 4 µg/mL-most cytotoxic*	Increase in ADAM10 enzyme activity (*4T1 or 4MTMHpc cell line, respectively)*: RT (55%) vs. RE + RT (74.5%) RE (43.3%) vs. RT (70.9%) vs. RE + RT (72.5%)	^60^Co γ-rays *45 Gy*	2016	[139]
Human lung squamous carcinoma H-520	RE *200 µg/mL*	RE significantly enhanced the radiosensitivity by inhibition of cellular proliferation, promotion of cell apoptosis and redistribution of cell cycle, possibly via deactivation of the Akt pathway	^60^Co γ-rays *1, 2, 4, 6, 8 and 10 Gy*	2010	[130]

ESCC = oesophageal squamous cell carcinoma; NSCLC = Non small cell lung cancer; RE = Recombinant Endostatin; RT = Radiotherapy.

**Table 2:** Endostatin combined with radiotherapy in vivo mouse models.

Cancer Type	E/RE *Dose*	Main Result	RT Type *Dose*	Year	Reference
LLC	RE *15 mg/kg*	Can promote the normalization of tumour blood vessels and increase the anti-tumour immune-related immune cells infiltrating the tumour post RT	Varian Clinac 600C (energy not specified, 6–10 MV X-rays) *10 Gy*	2020	[125]
EC	E *50 mg/kg*	Enhanced the anti-tumour effects of RT and prolonged disease-free survival	Cs^137^ *γ-rays* *Dose rate 6 Gy/min (dose not specified)*	2007	[135]
ESCC	RE *2.5, 5 and 10 mg/kg*	RE promotes the efficacy of RT on esophageal cancer, which may be partly realized by inhibiting the activity of VEGF related signal pathways	6 MV X-ray *10 Gy*	2016	[140]
NSCLC	RE *0.75 mg/mL for 7 days*	RT + weekly RE showed synergistic effects, produced by: RE’s stability, RE’s improvement of tumour hypoxia resulting in increased sensitivity to RT and RE’s inhibition of RT-induced tumour angiogenesis	6 MV X-ray *10 Gy*	2011	[144]
ESCC	RE *15 mg/kg*	RE + RT was more effective at delaying tumour growth than single therapy	RS2000 X-ray irradiator (kV range) *2, 4, 6 or 8 Gy*	2015	[141]
LLC	RE *0, 2.5, 5, 10, and 20 mg/kg*	RT + Endo + CP673451 treatment markedly inhibited tumour growth with no improvement in the overall survival and significantly reduced the tumour MVD	Varian Clinac 600C (6–10 MV X-rays) *12 Gy*	2018	[145]
HCC	RE *2, 4, 8, 16, and 32 mg/kg*	Combination therapy regulated the expression of genes controlling angiogenesis and cell adhesion. Synergistic effect of RE + RT against HCC in vivo and *in vitro*	6 MeV electron beam *10 Gy*	2017	[142]
NPC	RE *20 mg/kg/d*	RE normalized tumour vasculature, which alleviated hypoxia and caused significant radiosensitization in human NPC	160 kV X-ray *6 Gy*	2012	[127]
HNSSC	Endostatin *2.5 mg/kg/day*	Endostatin + RT produced an increase in cow pulmonary artery endothelial apoptosis compared with either treatment alone	not stated *15 Gy/day*	2000	[146]
Colorectal cancer	RE *20 mg/kg*	The tumour growth inhibition rate in the RT + RE treatment group > single therapy groups	6 MV X-ray *6 Gy*	2017	[143]
NPC	RE *20 mg/kg*	The tumour inhibition rates of RE, RT and RE + RT were 27.12, 60.45 and 86.11%, respectively. Tumour VEGF levels in the RE + RT group < RT only and control groups	5 MV X-ray *20 Gy*	2012	[136]
NPC/ ung adenocarcinoma	RE *20 mg/kg*	RE sensitized anti-tumour/anti-angiogenic RT effects by increasing apoptosis of the endothelial and tumour cells, decreasing hypoxia, and changing proangiogenic factors	6 MV X-rays *6 Gy per day to 30 Gy, once a day for 1 week*	2009	[147]

EC = epidermoid carcinoma; SSC = squamous-cell carcinoma; ESCC = oesophageal squamous cell carcinoma; LLC = Lewis lung carcinoma; HCC = Hepatocellular carcinoma; HNSCC = head and neck squamous cell carcinoma; NPC= Human nasopharyngeal carcinoma; NSCLC = Non-small cell lung cancer; RE = Recombinant Endostatin; RT = Radiotherapy; MVD=Microvessel Density.

### 3.3. Current Status of Clinical Trials in NSCLC Patients Investigating Radiotherapy Combined with RE 

Numerous clinical trials and meta-analyses have demonstrated a significant survival benefit with an acceptable safety profile when treating late-stage NSCLC patients with RE, including synergistic effects with concurrent chemoradiotherapy (CCRT), such as vinorelbine, platinum-based chemotherapy, docetaxel, and etoposide. These improvements were also seen in patients resistant to previous chemotherapy or patients with complete surgical resection [61,137,148,149,150,151,152,153,154,155,156]. Table 3 and the Supplementary material Table S1 provide a selected overview of clinical trials investigating RE combined with chemotherapy alone or with RT/CCRT, respectively. Already in 2005, RE was approved by CFDA in combination with vinorelbine/cisplatin for patients with advanced NSCLC [148]. The benefits of RE therapy have also been shown in NSCLC patients with bone metastasis [124,157]. Despite mostly positive findings, a few trials could not confirm a significant prolongation of the progression-free survival (PFS) and overall survival (OS) for RE combined with chemotherapy. In addition, no PFS benefit was shown in a multi-center phase II study in which 126 previously untreated advanced-stage NSCLC patients were enrolled and randomized to receive RE plus paclitaxel/carboplatin or paclitaxel/carboplatin alone [158]. 

RE combined with photon-based RT has also been studied extensively to validate its function as a hypoxic tumour radiosensitizer (Table 3). Multiple trials demonstrated a good short-term survival and response in non-resectable stage III NSCLC [124,159]. Interestingly, vascular normalization appeared approximately 1 week after administration of RE, opening an ideal time window for RT [160]. Recently, Yuan *et al.* clarified via a meta-analysis that the benefits of the addition of RE to CCRT in NSCLC are associated with a significantly higher ORR (objective response rate), disease control rate (DCR), and survival rate compared to CCRT, with similar incidences of main adverse events [149]. The pooled analysis of Zhang *et al.* concluded that RT combined with endostatin may be a promising strategy for locally advanced NSCLC patients with poor performance status who cannot tolerate chemotherapy [66]. However, the phase II study on RE in combination with paclitaxel, carboplatin, and RT in patients with unresectable NSCLC did not meet its goal without inducing unacceptable toxicity [161]. Continuous intravenous RE in combination with concurrent etoposide/cisplatin and RT resulted in a preferable OS, promising 2-year PFS with tolerable toxicities but did not prolong median PFS (HELPER study 2019) [159]. Interestingly, RE delivered by continuous intravenous pumping with CCRT may be a better option than intravenous injection in terms of potential survival and safety [162,163]. Recently, RE combined with whole-brain RT showed better survival and improved cerebral perfusion parameters in NSCLC patients with brain metastasis [164]. However, it should be noted that the number of patients in every single trial is too limited to achieve a definite conclusion.

Next to CCRT, immune checkpoint inhibitors (ICIs) and anti-angiogenic drugs are gaining momentum as a promising combined treatment strategy for NSCLC. Nivolumab, atezolizumab, and pembrolizumab have been approved as second-line treatments for advanced NSCLC [60]. Recently, the first study investigating the combination of RE with nivolumab showed a favourable efficacy and safety profile [60]. 

**Table 3:** A selection of clinical trials on RE combined with RT in NSCLC.

Cancer Type	Phase	E/RE *Dose*	Year	n	Combined Therapy	Overall Result	Reference
NSCLC	Pro cohort	RE *15 mg/day*	2012	25	RT	(+) short term therapeutic effects and local control rates. no severe adverse effects (-) no improvement of 1/3 year OS	[160]
NSCLC	n.s.	RE *15mg/day for 10 days*	2013		RT	(+) decreased hypoxia	[124]
BM of NSCLC	II	RE *7.5 mg/m^2^/day*	2014		RT	(+) can relieve brain oedema	[165] NCT01410370
Stage III NSCLC	SA pro II	RE *7.5 mg/m^2^/day for 7 days at week 1, 3, 5 and 7*	2015	48	RT/DOC and CIS	(+) promising survival and local control rates	[166] NCT01218594
Stage IIIA/B NSCLC	SA pro II	E *7.5 mg/m^2^ on day 1–14, every 3 weeks*	2016	19	RT/TC	(-) did not meet the goal per study design with unacceptable toxicity	[161] NCT01158144
Stage IIIA/B NSCLC	SA retro	RE *7.5 mg/m^2^/day for 7 days at week 1, 3, 5 and 7*	2020		CCRT	Inflammation-based factors as biomarker	[167]
Stage III NSCLC	SA pro II	RE *7.5 mg/m^2^/day, 14 days/cycle*	2019	67	RT/ *ETO-CIS*	(-) did not prolong median PFS (+) preferable OS, promising 2-year PFS with tolerable toxicities	[159] HELPER study NCT01733589
Stage III NSCLC	II	RE *7.5 mg/m^2^/day for seven day*	2020	48	IV RE + RT/DOC/ CIS vs. CIV RE + RT/ *ETO-CIS*	CIV > IV	[162]
Local aLSCC	retro	RE *7.5 mg/m^2^/day for 14 days (every 3 weeks)*	2020	94	RT/NP	Lipoprotein (a) as biomarker	[168]
Stage III NSCLC*	IV	RE *7.5 mg/m^2^/day, 14 days/cycle*	/	/	Durvalumab/ reduced-dose CCRT (50 Gy)	Not yet recruiting	NCT04613284
Stage III NSCLC	Multi-centre, prospective real-world study	RE *n.s.*	/	/	CCRT	Not yet recruiting	NCT04161352

(aNSCLC) advanced non-small cell lung cancer, (aLCC) advanced lung squamous carcinoma, (BM) brain metastasis, (CCRT) concurrent chemoradiotherapy, (CIS) Cisplatin, (CIV) Continuous intravenous pumping, (DOC) Docetaxel, (E) Endostatin, (ETO-CIS) etoposide-cisplatin, (IV) intravenous injection, (n) number of study participants, (n.s.) not specified, (NP) vinorelbine and cisplatin, (pro) prospective, (RE) Recombinant endostatin or endostar, (retro) retrospective, (SA) Single arm, (TC) paclitaxel-carboplatin, (*) who cannot tolerate 60 Gy RT.

## 4. Discussion and Concluding Remarks

Targeting angiogenesis as a tumour treatment strategy is undoubtedly complex, since pathological angiogenesis causes numerous changes in the TME. In addition, potential elevated toxicity in chemotherapeutic-anti-angiogenic combinations, and the impact of the normalization window and resulting oxygenation status on RT are still a topic of debate. To assess the ability of an additional variable to potentiate the effect of a treatment, it is of critical importance to first assess the impact of each individual treatment alone and understand the underlying biological mechanisms. To that end, photon-based RT as a standalone target against tumour angiogenesis has been shown to cause angiogenic stimulation when compared to proton therapy (PT) (Figure 1). There is an opportunity to take advantage of the vascular normalization window of 5–7 days upon RE administration, but the combination of RE with PT may cause a synergistic angiogenic suppression and the effect of such a combined treatment has not been assessed so far. Furthermore, an argument could also be made on the necessity to administer RE or to lower the dosage, since PT already boosts anti-angiogenic pathways. In addition, high-LET radiation, such as carbon ions, is less dependent on the oxygen effect to kill tumour cells, which questions the benefit of concurrent anti-angiogenic therapy [115,169]. 

NSCLC is known as a radioresistant cancer due to the presence of cancer stem cells, an epithelial-mesenchymal transition, and its high proportion of hypoxic cell populations [6,170]. RE has already been shown to act as a radiosensitizer in several cancer types (Table 2) and this led to several clinical trials (Table 3 and Supplementary Table S1). These clinical trials confirmed synergism when RE was combined with CCRT without causing major toxicities. However, more randomized controlled trials are needed to confirm long-term survival benefits [149]. In addition, while the majority of clinical trials apply similar RE dosages (Table 3 and Supplementary Table S1), the administration routes and timing varies, as well as the total length of the combined treatment cycles. It was out of the scope of the current work to compare administration routes and clinical details regarding patient selection and treatment evaluation, but it is important to keep into consideration that the dosage and timing of RE administration requires careful attention in both clinical and preclinical studies. Multiple short-term doses of anti-VEGF therapy could be required to generate a true long-term benefit and tumour regression. Unfortunately, prolonged exposure to anti-angiogenics results in increased hypoxia and systemic toxicity, compounding the dilemma that exists around the incorporation of anti-angiogenics with RT. Re-increased hypoxia and prolonged VEGF suppression, causing further local increases in hypoxia, are also coupled with pH shifts in the TME and consequent acidosis [171]. Furthermore, anti-angiogenic therapy has been shown to promote tumour metastasis. Due to hypoxic conditions, a pressure mechanism is generated that causes selectivity for tumour cells that harbour increased aggressiveness and lower sensitivity to anti-angiogenic therapy [172]. Work by Yang and co-workers also exhibited that anti-VEGF cessation-associated regrowth and remodelling of hepatic vasculatures provided a structural basis for cancer metastasis [173]. Additionally, a double-edged sword scenario appears when trying to resolve these complications with shortened anti-angiogenic treatment periods, as a relapse in pathological angiogenesis has been observed in multiple diseases after shorter treatment with anti-angiogenics or during drug holidays [174,175]. These preliminary conclusions provide reasons to motivate the use of RE in combination with particle therapy in future assessments. The inverted depth-dose profile of carbon-ion and protons allows dose sparing of organs at risk of co-irradiation, maximum dose deposition within the tumour, and the potential of dose escalation [176]. Several studies have also revealed previously unrecognized biological advantages of proton therapy (PT) specifically [177]. Moreover, the lower integral dose of PT and its dose sparing properties, have been found to reduce the exposure of circulating lymphocytes and the immune organs at risk compared to photon-based RT [178]. Preliminary findings of a study in NSCLC with underlying idiopathic pulmonary fibrosis showed a trend of non-statistically significant better OS compared to X-rays for patients treated with PT [179]. However, a randomized phase III clinical trial of intensity modulated photon therapy versus passive scattering proton therapy of locally advanced NSCLC, reported no benefit in the primary endpoints (grade 3 pneumonitis and local failure) after PT [180]. Furthermore, it is also important to note the difficulties in treating lung cancer with PT, largely due to the impact of highly heterogeneous tissues in the proton path on the proton dose distributions and respiratory motion during irradiations [181,182]. The higher RBE at the distal edge of the beam could potentially be problematic if this region is deposited in an organ at risk such as the heart. When one applies generous margins to circumvent the problem of tumour motion and tissue heterogeneity, this might counteract the dosimetric advantage and cause more normal tissue injury [178]. This adds an additional layer of complexity in the incorporation of proton therapy with RE seeing as microvascular heterogeneity is another important variable to consider due to its abundance in lung tissue [183], and its direct influence on the efficacy of anti-angiogenic therapy. However, the use of intensity modulated PT in combination with real-time volumetric image guidance, management of organ and tumour motion and accurate models which incorporate set-up uncertainties will assist to solve this problem [182].

Anti-VEGF therapy supresses neovascularization efficiently, whilst mature blood vessels are not as affected. This has been postulated to be attributable to a loss in dependence on growth factor signalling by mature vessels and potential anti-VEGF-A/VEGFR pathway therapy resistance as a consequence [108]. It is clear that the potential for improved therapeutic outcomes by combining RE and PT for the treatment of NSCLC is an avenue worth exploring, particularly in combination with immunotherapy. However, this will not only require a better understanding of the effects of PT on angiogenesis pathways, but also on the immunomodulatory effect of particles, such as protons and carbon ions. Immune checkpoint modulators such as anti-PD1 or anti-PDL1 agents are considered to be a breakthrough in the treatment of NSCLC [184]. The view that RT, and particularly particle therapy, can provoke a systemic immune response, provides a strong rationale for the combination with immunotherapy [185]. In this context, anti-angiogenic drugs, such as RE, could potentiate immunotherapy through vascular normalization and optimizing the tumour immune microenvironment. This rationale is currently accepted as a valid therapeutic strategy that can enhance cancer immunity, where the addition of RT could further expand the treatment landscape of NSCLC.

## Data Availability

Data sharing is not applicable.

## Supplementary Materials

The following supporting information can be downloaded at: https://www.mdpi.com/article/10.3390/ph16020219/s1, Table S1: Current status of clinical trials on endostatin combined with chemotherapy in NSCLC. References [186,187,188,189,190,191,192,193,194,195,196] are cited in the supplementary materials.
