# Supplementary material for: Recombinant Endostatin as a Potential Radiosensitizer in the Treatment of Non-Small Cell Lung Cancer
Source: Pharmaceuticals (Basel). 2023 Jan 31;16(2):219. doi: 10.3390/ph16020219 (PMC9961924; doi:10.3390/ph16020219)
Supplement: Supplementary file 1 [file pharmaceuticals-16-00219-s001.zip › pharmaceuticals-2141054-supplementary.pdf]

Table S1. Current status of clinical trials on endostatin combined with chemotherapy in NSCLC.

Disease type	Phase	E/RE<br>*Dose*	Combined<br>Therapy	Main result	Year	Reference
Positive results						
NSCLC	IV	RE<br>*7.5 mg/m²*<br>*d1–14*	NP	(+) Improved synergistic effect if concomitant	2012	[186]
stage IIIA (N2) NSCLC	IV	RE<br>*7.5 mg/m²*<br>*d1–14*	NP	(+) Combined therapy increased therapeutic efficacy without increasing adverse effects	2016	NCT02497118 [187]
aNSCLC	III	RE<br>*167.5 mg/m²*<br>*d1–14*	NP	(+) Significantly and clinically meaningful improvement in response rate	2005	#[154]
aNSCLC	III	RE<br>*7.5 mg/m²*<br>*d1–14*	NP	(+) Combined therapy can result in a significant clinical and survival benefit compared with NP alone	2013	[148]
aNSCLC	II	RE	NP	(+) Significant efficacy and safety if combined	2005	#[188]
aNSCLC		RE	TP/NP	(+) Combined therapy is effective and safe	2011	#[153]
NSCLC	single arm II	RE<br>*15 mg*<br>*d1-14*	CIS/ETO	(+) Combined therapy showed promising results: median PFS and OS were 5 and 11.5 months, respectively, and the ORR was 69.7%	2011	[151]
aNSCLC	II	RE<br>*7.5 mg/m²*<br>*d1–14 (3-week cycle)*	GEM/CIS	(+) Combined therapy improves objective response and may improve survival	2012	[189]
pre-treated aNSCLC	II	RE<br>*210 mg, every 4 weeks*	Nivolumab	Favourable efficacy and safety profile		[60]
aNSCLC		RE<br>*#*	DOC	(+) RE may prolong time to progression in patients that benefited from DOC without increased toxicities	2013	#[190]
aNSCLC		RE<br>*#*	CIS/ETO	(+) Combined regimen has better short-term effect and tolerance	2013	#[152]
aNSCLC		RE<br>*7.5 mg/m²*<br>*d1–14 (1 cycle)*	cisplatin	(+) RE administered 4 days before CT and combined from day 5 better than combined therapy from day 1	2015	[191]
m/aNSCLC		RE<br>*15 mg once every other day for 2 weeks (4x)*	apatinib mesylate vs paclitaxel	(+) Combined therapy showed a better therapeutic effect with improved immune resistance and less side effects	2019	[192]
NSCLC		RE<br>*#*	DOC/CB	(+) Combined therapy prolonged disease-free survival and improved three-year OS	2012	#[137]
BM of NSCLC		*RE*<br>*7.5 mg/m²*<br>*d1–14 every cycle*	NP	(+) higher ORR (30% vs. 0%), longer OS (21.44 ± 17.28 vs. 7.71 ± 4.68 months) when combined therapy	2016	[157]
aNSCLC	retro	RE<br>*15 mg*<br>*day 1 or 2*	CT	(+) IV and arterial infusion enhanced the PFS and OS without increasing the risk of toxicity	2015	[150]
aNSCLC	retro	E<br>*7.5 mg/m²*<br>*d1–14*	Emcitabine/CB/ GEM	(+) Combined therapy achieved a better disease control rate compared to CT only	2018	[193]
aNSCLC	Com	RE<br>*7.5 mg/m²*<br>*d1–14*	platinum based CT	(+) Combined therapy was more effective than CT alone if non-driver gene mutated	2019	[194]
Negative results						
ED-SCLC	II	RE<br>*7.5 mg/m²*<br>*d1–14 each*	ETO-CB	(-) Acceptable toxicity profile, but did not improve OS, PFS, and OR		[195]
aNSCLC	II	RE<br>*7.5 mg/m²*	TC	(-) Good safety profile but differences in PFS and OS not significant		[158]

		d8-21 each cycle			
aNSCLC	retro	RE<br>*7.5 mg/m²*<br>*d1–14 every 3 weeks*	PEM/CIS	(-) Combined therapy did not prolong PFS or OS, but a trend of improved PFS was observed in patients administered RE+PEM	[196]
Ongoing					
sq-NSCLC	II	RE<br>*210 mg*<br>*d1-3 every 3 weeks*	envafolimab/platinum-based CT	Recruiting	NCT05243355
driver gene negative aNSCLC	II	RE<br>*210 mg*<br>*d1 every 3 weeks*	platinum-based CT	Recruiting	NCT05574998
aNSCLC		RE<br>*15 mg/m²*<br>*d1-5 (every 3 weeks)*	PEM/ platinum-based CT	Not yet recruiting	NCT04094909
phase IB NSCLC	III	RE<br>*7.5 mg/m²*<br>*d1–14*	PEM/DOC/ CIS	Status: unknown	NCT02001168
driver gene negative aNSCLC	II	RE<br>*210 mg*<br>*d1 (every 3 weeks)*	PD-1 mAb/ platinum-based CT	Not yet recruiting	NCT05448781
aNSCLC	II	RE<br>*210 mg*<br>*d1-7 (every 3 weeks)*	Envafolimab/ S-1	Not yet recruiting	NCT05529355
a/metNSCLC	I/ PK study	RE*<br>*Cycle 1: 7.5 mg/m²*<br>*d1–14*<br>*Cycle 2-4: 105 mg/m²*	platinum-based CT	Not yet recruiting	NCT04942301

(aNSCLC) advanced non-small cell lung cancer, (BM) brain metastasis, (CB) carboplatin, (CECs) circulating endothelial cells, (COM) comparative study, (CCRT) concurrent chemoradiotherapy, (CIS) cisplatin, (CIV) continuous intravenous pumping, (CT) chemotherapy, (DOC) docetaxel, (ETO) etoposide, (ED-SCLC) extensive disease small cell lung cancer, (GEM) gemcitabine , (IV) intravenous injection, (KDR) kinase insert domain receptor, (met) metastatic, (M2ES) polyethylene glycol rh recombinant endostatin, (OS) overall survival, (ORR) objective response rate, (PD-1) programmed cell death protein 1, (PEM) pemetrexed, (PFS) progression free survival, (PK) pharmacokinetic, (VEGFR2) vascular endothelial growth factor receptor 2, (NP) vinorelbine and cisplatin, (mNSCLC-aNSCLC) middle or advanced non-small cell lung cancer, (retro) retrospective study, (TC) paclitaxel-carboplatin, (TP) paclitaxel plus cisplatin/carboplatin, (#) Article in Chinese, (*) intravenous infusion and continuous (pump) infusion.
